# Identification of 4-hydroxyphenethyl alcohol and 6-hydroxynicotinic acid as novel spoilage biomarkers in refrigerated beef

**DOI:** 10.1016/j.fochx.2026.104549

**Published:** 2026-09-30

**Authors:** Chuan Liu, Nan Liu, Juan Zhang, Linlin Wang, Chunjiao Bao, Hao Liu, Wei Zhang, Lirong Sun, Ailiang Chen

**Affiliations:** a College of Food Science and Technology, Hebei Agricultural University, Baoding, Hebei, China; b State Key Laboratory for Quality and Safety of Agro-Products, Institute of Quality Standards and Testing Technology for Agro-Products, Chinese Academy of Agricultural Sciences, Beijing 100081, China; c Tongliao Agricultural and Livestock Product Quality and Safety Center, Tongliao 028000, China; d Hebei Key Laboratory of Analysis and Control of Zoonotic Pathogenic Microorganism, Hebei Agricultural University, Baoding, Hebei, China

**Keywords:** Refrigerated beef, Quality deterioration, Novel biomarkers, Freshness monitoring

## Abstract

Traditional freshness indices, including total viable count (TVC) and total volatile basic nitrogen (TVB-N), have limitations in elucidating the underlying molecular mechanisms of meat spoilage. This study investigated changes in beef freshness indices during refrigerated storage at 4 °C and determined that beef entered the spoilage stage on day 7. Untargeted metabolomics characterized metabolic changes and revealed two candidate biomarkers, 4-hydroxyphenethyl alcohol and 6-hydroxynicotinic acid. On days 7 and 11, the relative abundance of 4-hydroxyphenethyl alcohol was 28.88 and 115.20 times that on day 0, respectively, whereas that of 6-hydroxynicotinic acid was 33.47 and 66.66 times. Targeted quantification showed that 4-hydroxyphenethyl alcohol reached 1025.69 μg/kg on day 7 and 5081.85 μg/kg on day 11, while 6-hydroxynicotinic acid reached 685.18 μg/kg on day 7 and 1368.12 μg/kg on day 11. These biomarkers provide promising targets for spoilage warning, rapid freshness assessment, and shelf-life prediction of refrigerated beef.

## Highlights

• Untargeted and targeted metabolomics profiling of beef quality changes during refrigerated storage.
• Fourteen metabolites showed strong positive correlations with key freshness indicators.
• 4-Hydroxyphenethyl alcohol and 6-hydroxynicotinic acid were identified as novel biomarkers of beef spoilage.
• Targeted quantification confirmed accumulation of both biomarkers at the critical spoilage point.

## Graphical abstract

**Figure:**
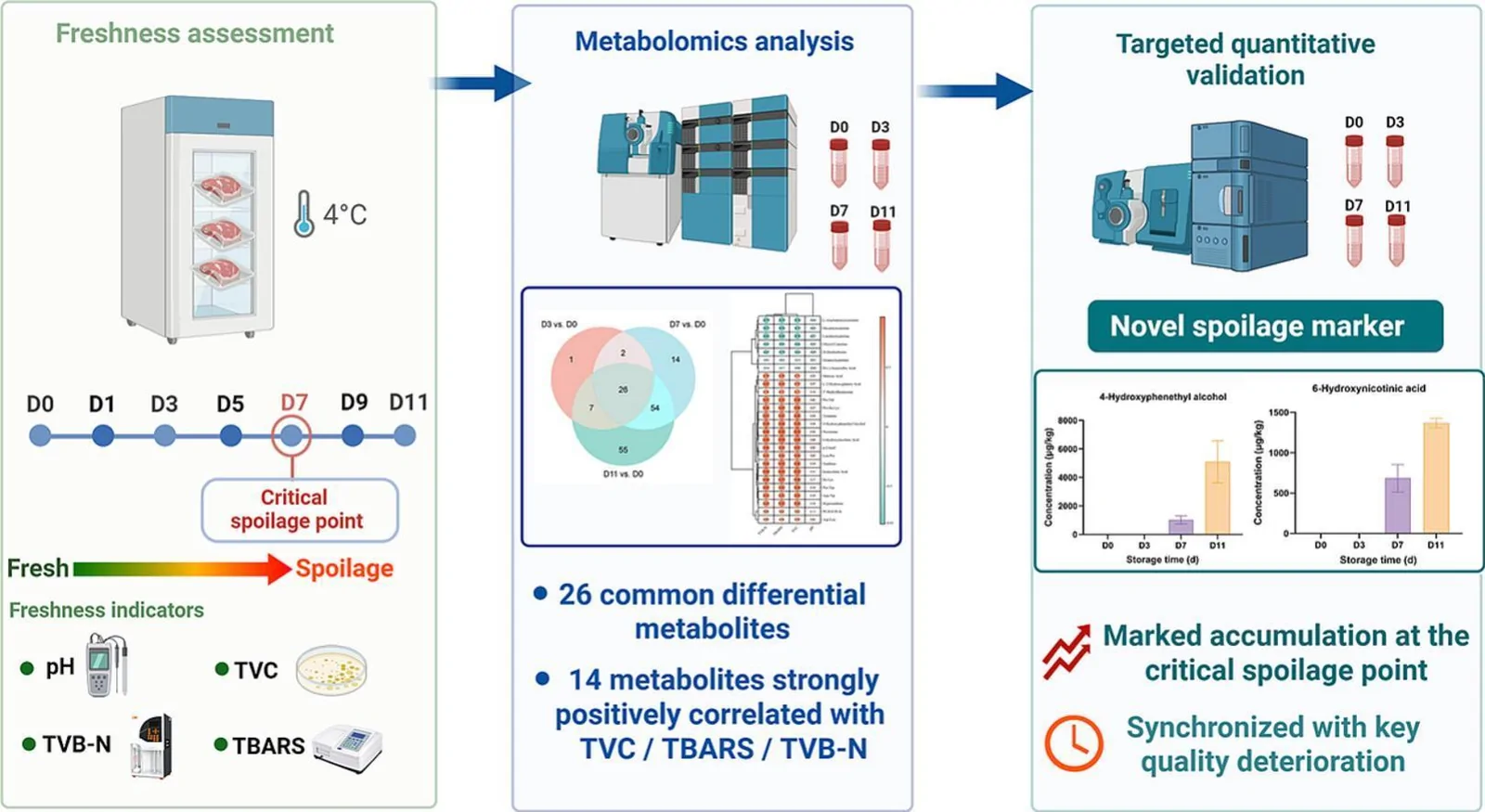

## 1 Introduction

Beef is rich in nutritional components such as high-quality protein and unsaturated fatty acids and has high nutritional and commercial value, making it one of the most widely consumed meats worldwide (Ponnampalam et al., 2024). Compared with frozen beef, refrigerated beef generally better preserves its original color, texture, flavor, and nutritional quality, thereby meeting consumers' demands for the palatability and eating quality of “fresh meat” (Coombs, Holman, Friend, & Hopkins, 2017). However, during slaughtering and processing, transportation, retail display, and household storage, beef is inevitably affected by microbial growth and endogenous enzymatic activity. As storage time increases, proteins undergo gradual degradation into peptides, free amino acids, and nitrogen-containing metabolites (Triki, Herrero, Jiménez-Colmenero, & Ruiz-Capillas, 2018). Meanwhile, lipid oxidation generates aldehydes, ketones, and acids, thereby further contributing to the decline in the nutritional value and sensory quality of beef. The accumulation of various small-molecule metabolites during storage can objectively reflect the extent of beef quality deterioration. Therefore, accurately identifying potential biomarker compounds based on changes in metabolites during storage is of great significance for the precise monitoring of beef quality.

Traditional methods for freshness assessment mainly include sensory evaluation and the determination of biological and chemical indicators. However, these methods are characterized by a high degree of subjectivity and limited sensitivity, making it difficult to accurately identify changes in specific metabolites and reveal meat quality deterioration at the molecular level (Chen et al., 2024). In contrast, metabolomics is an analytical approach that systematically characterizes and quantifies small-molecule metabolites in biological samples, thereby enabling a comprehensive understanding of metabolic changes under specific physiological states or storage conditions (Lin et al., 2024). Previous studies have shown that, during the refrigerated storage of pork and poultry meat, untargeted metabolomics can effectively identify differential metabolite profiles between fresh and spoiled meat. Some of these differential metabolites are significantly correlated with meat quality indicators and may serve as potential biomarkers for freshness assessment (Zhang et al., 2020; Zhao et al., 2022). In addition, metabolomics enables the identification and classification of a large number of metabolites, thereby facilitating the elucidation of key metabolic pathways, the construction of metabolic networks, and the inference of metabolite functions (Jia et al., 2021).

On this basis, this study employed a strategy combining freshness indicator monitoring with metabolomics to systematically characterize metabolic changes in beef during refrigerated storage and to elucidate the intrinsic correlations between differential metabolites and beef freshness deterioration. For the two screened metabolites, 4-hydroxyphenethyl alcohol and 6-hydroxynicotinic acid, a targeted quantitative method based on UPLC–QQQ–MS/MS was further established for absolute quantification. This study aimed to identify stable and reliable novel biomarkers for beef freshness assessment, thereby providing a theoretical basis and technical support for quality monitoring and spoilage early warning during the cold-chain storage of fresh beef.

## 2 Materials and methods

### 2.1 Experimental design

Beef samples used in this study were obtained from a slaughterhouse in Horqin District, Tongliao City, Inner Mongolia Autonomous Region, China. Fresh hindquarter meat from eight Simmental cattle from the same slaughter batch was selected as the experimental material. The hindquarter meat from each animal was divided into seven portions corresponding to storage days 0, 1, 3, 5, 7, 9, and 11, yielding a total of 56 samples. Each portion was individually sealed in a polyethylene bag and stored at 4 °C in a normal atmosphere. At each storage time point, eight samples, one from each animal, were used as independent biological replicates (*n* = 8) for the determination of pH, TVC, TVB-N, and TBARS. Samples collected on days 0, 3, 7, and 11 were further subjected to metabolomics analysis and designated as D0, D3, D7, and D11, respectively.

### 2.2 Determination of freshness indicators

#### 2.2.1 pH and TVC

A portable pH meter was used to determine pH. Before use, the electrode was calibrated with standard buffer solutions (pH 4.00, 7.00, and 9.00). The electrode was then inserted approximately 3 cm into the beef sample, and pH values were recorded from at least three different locations. TVC was assessed by bacterial plate enumeration. 10.0 g of beef sample was placed into a sterile bag containing 90 mL of sterile 0.9% NaCl solution for homogenization. The homogenate was then serially diluted, and 1 mL of an appropriate dilution was inoculated onto plate count agar. The plates were incubated at 36 °C for 48 h, after which colonies were counted, and the results were expressed as log CFU/g.

#### 2.2.2 TVB-N and TBARS

TVB-N was determined according to the Chinese National Standard GB 5009.228-2016, National Food Safety Standard—Determination of Volatile Basic Nitrogen in Foods. Briefly, 10.0 g of minced beef sample was placed in a distillation tube, mixed with 75 mL of distilled water, and allowed to stand for 30 min after thorough mixing. Subsequently, 1.0 g of MgO was added, and the sample was immediately analyzed using the automatic Kjeldahl apparatus. TBARS was determined according to the method described by Adame et al. (2024). 5 g of beef samples were homogenized after being mixed with 25 mL of trichloroacetic acid solution containing 0.1% EDTA. After that, 5 mL of the filtrate was mixed with 5 mL of a 0.83 mg/mL TBARS solution, and the mixture was heated at 80 °C in a water bath for 40 min to develop color. The mixture was then vortexed for 5 min at 4000 rpm. A 5 mL solution of trichloroacetic acid was used as a control. Finally, the absorbance was measured at 532 nm using a UV spectrophotometer. The results were expressed as mg MDA equivalents per kg of beef sample.

### 2.3 Untargeted metabolomics analysis

A 150 mg portion of each sample was mixed with 1 mL of methanol/water solution (1:1, v/v). The mixture was ultrasonically extracted for 10 min, vortex-mixed for 5 min, and then centrifuged at 14,500×*g* for 20 min. The supernatant was collected, filtered, and transferred into an injection vial for metabolomics analysis. Metabolomics data were acquired using a UHPLC-QTOF-MS platform consisting of an ExionLC system coupled to a TripleTOF 6600 mass spectrometer (SCIEX, USA). Chromatographic separation was achieved with a ZORBAX Eclipse C18 column (150 mm × 3.0 mm, 1.8 μm; Agilent, USA). The column was maintained at 40 °C, with the flow rate and injection volume set to 0.3 mL/min and 5 μL, respectively. For positive-ion acquisition, water containing 0.1% formic acid was used as mobile phase A, and methanol containing 0.1% formic acid was used as mobile phase B. For negative-ion acquisition, mobile phases A and B consisted of water and methanol containing 0.1% ammonium acetate, respectively. The gradient elution program was as follows: 0–1 min, 10% B; 1–8 min, 90% B; 8–12 min, 90% B; 12–12.1 min, 10% B; 12.1–17 min, 10% B. The MS parameters were as follows: spray voltage, 5500 V/−4500 V in positive/negative ion mode; declustering potential, 80 V; collision energy, 10 eV for MS1 and 35 ± 15 eV for MS2; scan ranges, 100–1000 Da for MS1 and 50–1000 Da for MS2; ion source temperature, 500 °C; curtain gas, 35 psi; and Gas 1/Gas 2, 50 psi.

### 2.4 Targeted quantification

The samples used for targeted quantitative analysis were obtained from the same storage experiment as those used for untargeted metabolomics analysis. The samples were extracted using the same procedure to obtain methanol/water (1:1, v/v) extracts. Subsequently, 100 μL of the extract was mixed with 150 μL of 0.1% formic acid aqueous solution. Analysis was performed using a UPLC-QQQ-MS system (ACQUITY UPLC, Waters, USA; Triple Quad 4500, SCIEX, USA). Chromatographic separation was carried out on a ZORBAX Eclipse C18 column (100 mm × 2.1 mm, 1.8 μm; Agilent, USA). Authentic standards of 6-hydroxynicotinic acid and 4-hydroxyphenethyl alcohol were purchased from Alta Scientific Co., Ltd. (Tianjin, China). 6-Hydroxynicotinic acid was analyzed in positive ion mode, with water containing 0.1% formic acid as mobile phase A and acetonitrile as mobile phase B. The gradient elution program was as follows: 0–0.50 min, 10% B; 0.50–5.50 min, 90% B; 5.50–9.00 min, 90% B; 9.00–9.10 min, 10% B; and 9.10–13.00 min, 10% B. 4-Hydroxyphenethyl alcohol was analyzed in negative ion mode, with 10 mM ammonium acetate aqueous solution as mobile phase A and acetonitrile as mobile phase B. The gradient elution program was as follows: 0–0.5 min, 10% B; 0.5–3.0 min, 30% B; 3.0–5.5 min, 90% B; 5.5–9.0 min, 90% B; 9.0–9.1 min, 10% B; and 9.1–13.0 min, 10% B. Key MS parameters were optimized to obtain optimal responses, as shown in Table 1. Calibration curves were constructed using compound concentration (x, μg/L) and peak area (y). The limits of detection (LOD) and limits of quantification (LOQ) were determined using the signal-to-noise ratio (S/N) approach, with S/N > 3 and S/N > 10 used as the criteria for LOD and LOQ, respectively.

**Table 1:** MRM ion pairs and mass spectrometric parameters of target metabolites.

Q1 Mass (Da)	Q3 Mass (Da)	Time (ms)	ID	DP	CE
140.2	122.1	80.0	6-Hydroxynicotinic acid	80.0	28.0
140.2	94.1	80.0	6-Hydroxynicotinic acid	80.0	30.0
137.0	119.0	120.0	4-Hydroxyphenethyl alcohol	−30.0	−80.0
137.0	106.2	120.0	4-Hydroxyphenethyl alcohol	−30.0	−80.0

### 2.5 Data analysis

Physicochemical indicators were measured with eight biological replicates, and the results are expressed as the mean ± standard deviation. Statistical significance was assessed using one-way ANOVA followed by Tukey's HSD multiple comparison test (*P* < 0.05). Raw metabolomics data were imported into MS-DIAL for preprocessing, including peak detection, mass calibration, and peak alignment. Metabolite annotation was performed based on accurate *m*/*z* values and MS/MS fragmentation spectra, with metabolite information matched against the Massbank and HMDB databases. Relative metabolite abundances were determined based on peak areas after data preprocessing. Orthogonal partial least squares discriminant analysis (OPLS-DA) was performed using MetaboAnalyst 6.0. Differential metabolites were screened based on variable importance in projection (VIP) > 1, Student's *t*-test (*P* < 0.05), and fold change (FC ≥ 1.5 or FC ≤ 0.67). Metabolites meeting these criteria were included in Table S1. Univariate receiver operating characteristic (ROC) curve analysis was performed for each candidate metabolite to evaluate its ability to discriminate D0 samples from D3, D7, and D11 samples, and the area under the curve (AUC) was used to assess discriminatory performance.

## 3 Results and discussion

### 3.1 Determination of beef freshness during refrigerated storage

To clarify the spoilage progression of beef during refrigerated storage at 4 °C in this study, four quality indicators, including pH, TVC, TVB-N, and TBARS, were continuously monitored. Bacterial proliferation is the primary cause of rapid quality deterioration in beef. TVC increased steadily from an initial value of 3.58 log CFU/g, reaching 6.26 log CFU/g on day 7 (Fig. 1A), exceeding the microbial acceptability limit for fresh meat (6 log CFU/g) (GB 4789.2-2016) (Huang et al., 2026). In addition, extensive microbial proliferation accelerates the degradation of muscle proteins, leading to a simultaneous increase in TVB-N and pH. The initial TVB-N value of beef was relatively low, but it rose to 17.79 mg/100 g on day 7 (Fig. 1B), exceeding the limit of 15 mg/100 g for fresh meat stipulated by the national standard GB 2707-2016 (Liu et al., 2026). The pH of beef gradually increased from 5.41 at the initial stage to 5.83 on day 11 (Fig. 1C), which was mainly attributed to the continuous accumulation of alkaline nitrogenous compounds, such as ammonia and biogenic amines, produced by protein degradation. During refrigerated storage, beef underwent continuous lipid oxidation, and TBARS was used as an indicator to evaluate the extent of lipid oxidation (Wu et al., 2026). The TBARS value gradually increased from 0.25 mg/kg at the beginning of storage to 1.0 mg/kg on day 11 (Fig. 1D).

**Fig. 1:**
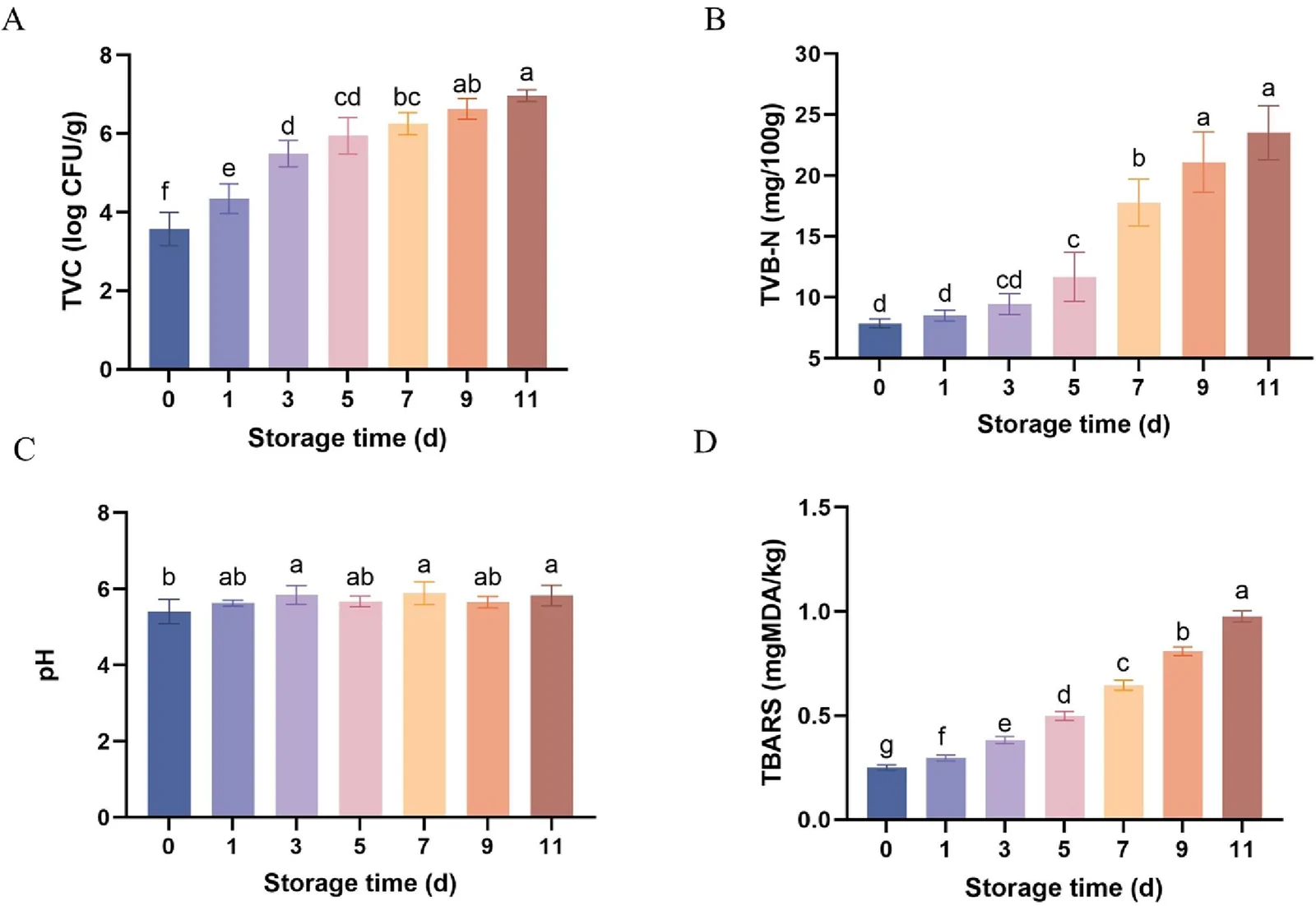
Changes in beef freshness indicators during refrigerated storage at 4 °C. (A) TVC. (B) TVB-N. (C) pH. (D) TBARS. Data are presented as mean ± SD. Different lowercase letters indicate significant differences among storage times (*P* < 0.05).

Based on the changing trends of the four indicators and their corresponding threshold values, both TVC and TVB-N exceeded the critical limits for freshness assessment on day 7, while pH and TBARS showed a continuous deterioration trend. Therefore, under the experimental conditions of this study, beef stored at 4 °C had entered a clearly identifiable spoilage stage by day 7, and its shelf life should not exceed 7 days. The determination of this time point also provided a basis for the validity of the subsequent metabolomics sampling points.

### 3.2 Untargeted metabolomics analysis

To investigate the effects of different refrigerated storage times on metabolic profiles, OPLS-DA models were constructed for pairwise comparisons of D3 vs. D0, D7 vs. D0, and D11 vs. D0, respectively. The score plots revealed complete separation of samples collected at each storage time point from the D0 group along the first predictive component, t[1], indicating significant differences in metabolic profiles (Fig. 2A–C). For the D3 vs. D0 model, R^2^Y and Q^2^ were 0.996 and 0.880, respectively; for the D7 vs. D0 model, R^2^Y and Q^2^ were 0.982 and 0.901, respectively; and for the D11 vs. D0 model, R^2^Y and Q^2^ were 0.996 and 0.965, respectively. In addition, 1000 permutation tests yielded *p* values <0.001 for all three models, indicating good model fit and predictive performance, with no evident overfitting (Fig. S1A–C). Differential metabolites were screened using the criteria of *P* < 0.05, FC ≥ 1.5 or FC ≤ 0.67, and VIP > 1, and volcano plots were used for visualization. The results showed that the number of differential metabolites generally increased with prolonged refrigerated storage. Compared with the D0 group, 20 upregulated and 16 downregulated metabolites were detected in the D3 group, 70 upregulated and 26 downregulated metabolites were detected in the D7 group, and 113 upregulated and 29 downregulated metabolites were detected in the D11 group (Fig. 2D–F). Venn diagram analysis identified 26 common differential metabolites shared among the three pairwise comparisons (Fig. 3A). Finally, based on the above screening criteria, a total of 159 differential metabolites were selected (Table S1).

**Fig. 2:**
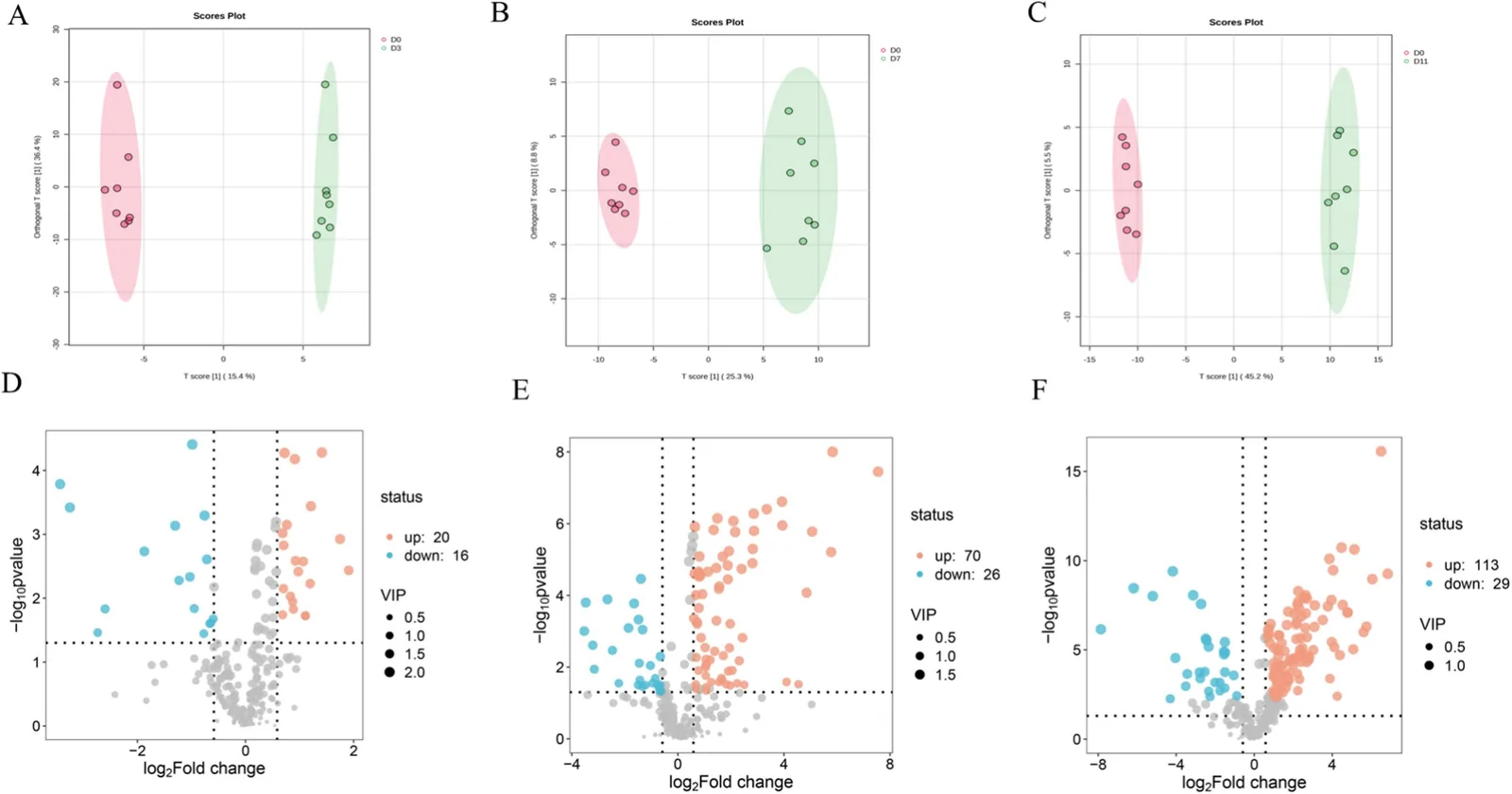
Differential metabolite analysis of beef at different refrigerated storage times (0, 3, 7, and 11 d). (A–C) OPLS-DA score plots for D3 vs. D0, D7 vs. D0, and D11 vs. D0, respectively. (D–F) Volcano plots for D3 vs. D0, D7 vs. D0, and D11 vs. D0, respectively.

**Fig. 3:**
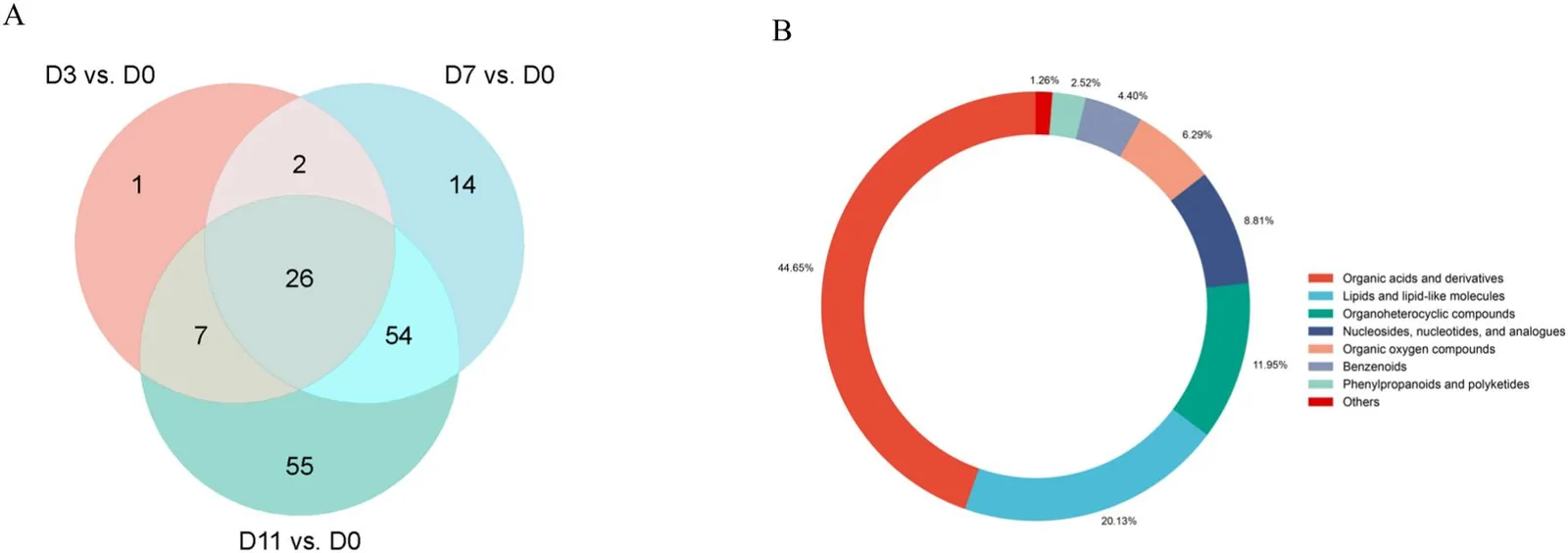
Venn diagram and classification composition of differential metabolites in beef during refrigerated storage. (A) Venn diagram of differential metabolites in the comparison groups D3 vs. D0, D7 vs. D0, and D11 vs. D0. (B) Classification and relative proportions of differential metabolites.

These differential metabolites were mainly annotated as organic acids and derivatives, lipids and lipid-like molecules, organoheterocyclic compounds, and nucleosides, nucleotides, and analogues, among other categories (Fig. 3B). Organic acids and derivatives were the predominant class of differential metabolites. Beef inherently contains various endogenous proteases, which are rapidly activated immediately post-slaughter and retain considerable activity even under refrigerated conditions, thereby promoting the gradual degradation of proteins into small peptides and free amino acids. The marked upregulation of these metabolites during the middle and late stages of storage, namely days 7 and 11, indicated that the samples had entered the spoilage phase (Wang et al., 2026). Among them, the accumulation of free amino acids, such as proline, phenylalanine, and methionine, as well as some small peptides, is closely associated with the development of bitterness in beef (Wang et al., 2026). Amino acids not only serve as key nitrogen sources for microbial proliferation and metabolism but also act as major precursors of typical spoilage metabolites, such as ammonia, biogenic amines, and various volatile organic compounds. During storage, a decrease in tyrosine content is often accompanied by a marked accumulation of tyramine (Chen et al., 2025; Wang et al., 2025). Within the organic acids and derivatives category, malonic acid showed the most pronounced change in abundance. Excessive accumulation of organic acids is a major factor contributing to the intensified sour taste of spoiled beef. The second major category was lipids and lipid-like molecules, whose changes were associated with lipid oxidation and membrane damage. Most free fatty acids showed an increasing trend during the 11-day refrigerated storage period. The accumulation of free fatty acids can further promote lipid oxidation and generate volatile compounds, such as aldehydes and alcohols, thereby accelerating the development of off-flavors (Cao et al., 2026). Nucleosides, nucleotides, and analogues, as key physiologically active components involved in metabolic processes, play an important role in maintaining beef freshness. Inosine monophosphate (IMP) is an important flavor-active compound in meat and a key indicator for freshness assessment. The abundance of IMP decreased on days 7 and 11 of refrigerated storage, whereas its degradation products, hypoxanthine and xanthine, both of which belong to the organoheterocyclic compounds category, accumulated continuously with prolonged storage. The accumulation of these two compounds was closely associated with increased bitterness and aggravated spoilage (Liu et al., 2026). 6-Hydroxynicotinic acid, which belongs to the same category, was significantly upregulated on days 7 and 11 of refrigerated storage compared with the fresh samples (D0). In addition to organoheterocyclic compounds, 4-hydroxyphenethyl alcohol in the benzenoids category also showed significant differences in abundance during the middle and late stages of storage compared with the fresh samples. These changes in metabolite abundance may be closely related to the physiological metabolism of bacteria under stressful storage conditions.

### 3.3 Correlation analysis of differential metabolites with freshness indicators

Venn diagram analysis identified 26 differential metabolites shared across different refrigerated storage stages. Because these metabolites showed differential abundance at each storage stage, they may serve as candidate discriminative biomarkers for distinguishing different freshness states of beef. Spearman correlation analysis was further performed to evaluate their relationships with beef freshness indicators. These metabolites could be categorized into two correlation patterns (Fig. 4).

**Fig. 4:**
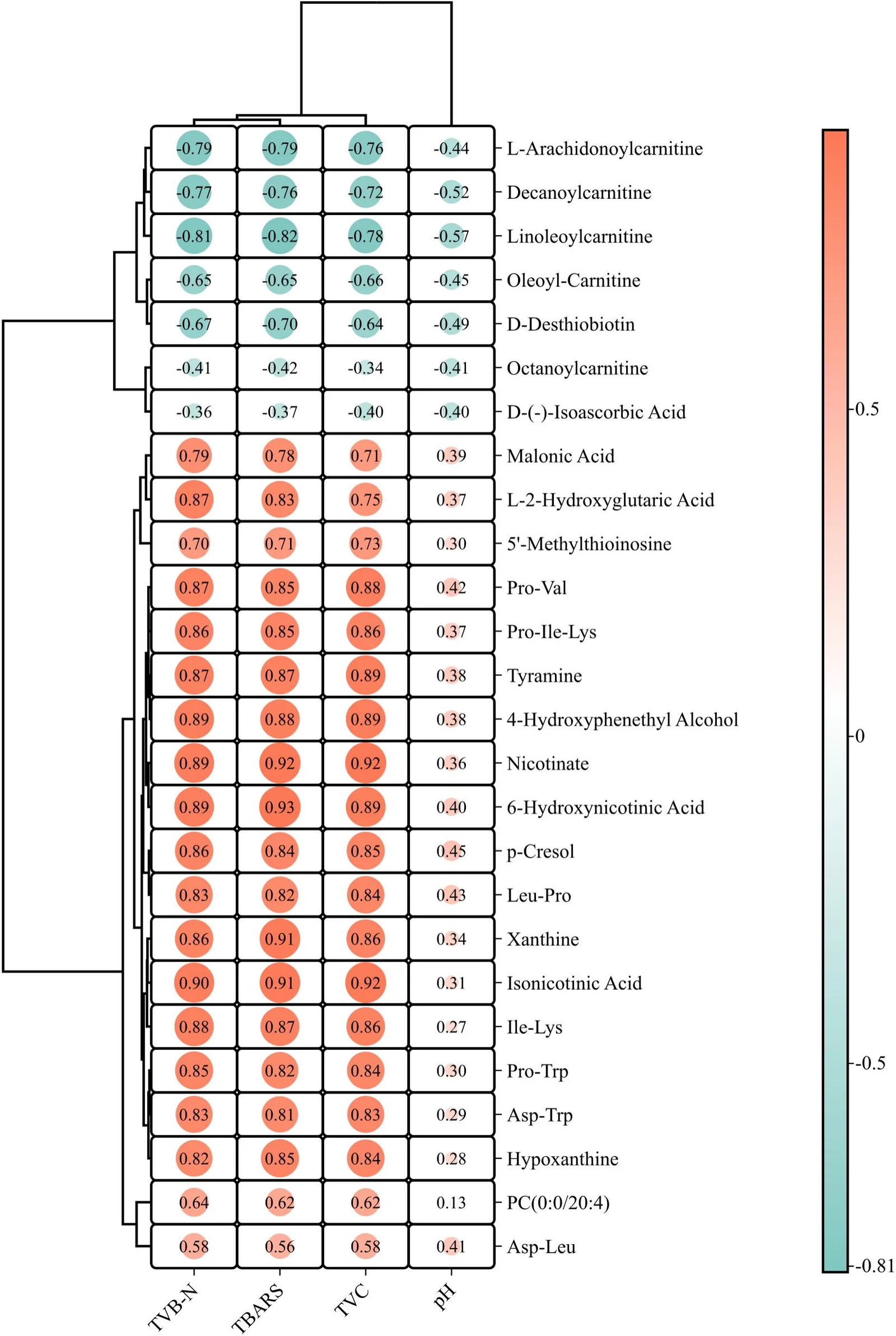
Correlation heatmap between freshness indicators (pH, TVC, TVB-N, and TBARS) and 26 differential metabolites.

The first group of metabolites, which included various acylcarnitines, was predominantly negatively correlated with TVC, TBARS, and TVB-N. Acylcarnitines are essential transport forms for the entry of fatty acids into mitochondria for β-oxidation and play an important role in lipid energy metabolism.

The second group of metabolites was positively correlated with TVC, TBARS, and TVB-N. Among them, hypoxanthine, isonicotinic acid, xanthine, p-cresol, 6-hydroxynicotinic acid, nicotinate, 4-hydroxyphenethyl alcohol, tyramine, and six peptides (Asp-Trp, Pro-Trp, Ile-Lys, Leu-Pro, Pro-Val, and Pro-Ile-Lys) exhibited strong positive correlations with TVB-N, TVC, and TBARS values (*r* > 0.80). The accumulation of these metabolites was closely associated with microbial decomposition activities and muscle autolysis. Specifically, hypoxanthine and xanthine are formed through the combined action of autolytic nucleotide degradation and bacterial activity, and are recognized as classical indicators of meat freshness decline. The elevation of nicotinic acid-related compounds, including nicotinate, isonicotinic acid, and 6-hydroxynicotinic acid, may be associated with microbial metabolism and the degradation of nicotinamide-containing coenzymes and related substances (Wang, Weng, et al., 2026; Wang, Zhang, et al., 2026; Yi et al., 2024; You et al., 2026). Tyramine, a typical biogenic amine, is an important alkaline nitrogenous product formed during meat spoilage and a major component of TVB-N. It is generated through the decarboxylation of tyrosine catalyzed by microbial tyrosine decarboxylase. In addition, other aromatic metabolites, including p-cresol and 4-hydroxyphenethyl alcohol, are also derived from the microbial degradation of aromatic amino acids. The six peptides were detected in substantial amounts on days 7 and 11 of refrigerated storage, which could be attributed to the combined action of endogenous and microbial proteases during the late storage period. Changes in these metabolites were closely associated with the spoilage progression of beef during refrigerated storage and, consequently, exhibited strong positive correlations with various freshness indicators.

### 3.4 Screening of novel spoilage biomarkers

To precisely identify key biomarkers that can indicate the transition of beef from freshness to spoilage based on the overall metabolic profiles, a multi-layer screening strategy was employed in this study. First, Spearman correlation analysis was performed between the 26 common differential metabolites and quality indicators, including TVC, TBARS, and TVB-N. Using the criteria of a correlation coefficient *r* > 0.80 and significant correlations with all three indicators (*P* < 0.05), 14 highly correlated metabolites were preliminarily identified, including hypoxanthine, isonicotinic acid, xanthine, p-cresol, 6-hydroxynicotinic acid, nicotinate, 4-hydroxyphenethyl alcohol, tyramine, and six peptides. Among the 14 candidate metabolites, the most pronounced increases were observed for 4-hydroxyphenethyl alcohol (28.88- and 115.20-fold), tyramine (57.06- and 90.87-fold), and 6-hydroxynicotinic acid (33.47- and 66.66-fold) at D7 and D11, respectively (Fig. 5A).

**Fig. 5:**
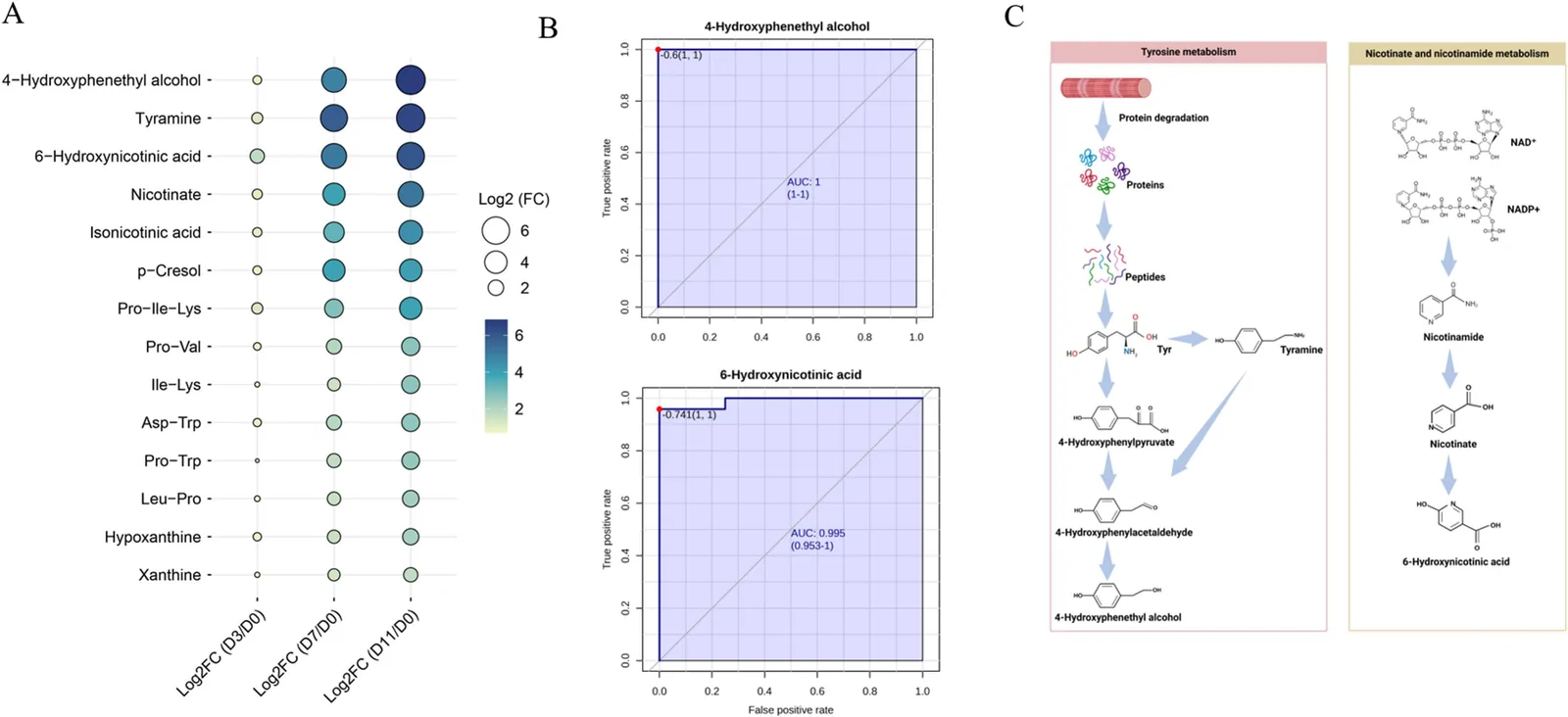
(A) Fold changes of 14 strongly correlated metabolites. (B) ROC curves of 4-hydroxyphenethyl alcohol and 6-hydroxynicotinic acid. (C) Proposed metabolic pathways for the formation of 4-hydroxyphenethyl alcohol and 6-hydroxynicotinic acid.

To further evaluate the ability of these candidate metabolites to discriminate between fresh and spoiled beef, univariate ROC analysis was performed for each of the 14 candidate metabolites (Figs. 5B and S2). The results showed that the AUC values of all 14 candidate metabolites were greater than 0.90, ranging from 0.922 to 1.000, indicating that these candidate metabolites all exhibited good discriminatory ability. Among them, 4-hydroxyphenethyl alcohol had the highest AUC of 1.000, while 6-hydroxynicotinic acid had an AUC of 0.995; the two metabolites exhibited the highest discriminatory performance among the 14 candidate metabolites. Based on the combined results of correlation analysis, ROC curve analysis, and metabolite accumulation characteristics, 4-hydroxyphenethyl alcohol and 6-hydroxynicotinic acid were ultimately selected as candidate biomarkers for beef spoilage.

During refrigerated storage, endogenous and microbial proteases in meat can promote the degradation of muscle proteins, such as myofibrillar proteins, generating protein fragments, peptides, and free amino acids (Peng et al., 2026). Tyrosine showed a slight increase during the early stage of refrigerated storage, possibly due to the release of free tyrosine through protein hydrolysis. Its relative abundance then gradually decreased from 1.22-fold at D3 to 0.34-fold at D11. Tyrosine, as one of the aromatic amino acids, may participate in the formation of 4-hydroxyphenethyl alcohol through two possible pathways. On one hand, tyrosine can be converted to 4-hydroxyphenylpyruvate via transamination, which is subsequently decarboxylated to 4-hydroxyphenylacetaldehyde and then reduced to 4-hydroxyphenethyl alcohol by alcohol dehydrogenase (Satoh, Tajima, Munekata, Keasling, & Lee, 2012). On the other hand, tyrosine can also be decarboxylated to tyramine, which is further oxidatively deaminated to 4-hydroxyphenylacetaldehyde, ultimately yielding 4-hydroxyphenethyl alcohol upon reduction (Fig. 5C) (Chen et al., 2019). Tyramine and 4-hydroxyphenethyl alcohol continuously accumulated during refrigerated storage. The changes in these metabolites were consistent with the proposed pathway for the conversion of tyrosine to 4-hydroxyphenethyl alcohol.

Meanwhile, nicotinamide coenzymes such as NAD^+^ and NADP^+^ may undergo degradation or metabolic conversion during refrigerated storage, releasing nicotinamide. Nicotinamide can be hydrolyzed to nicotinate by nicotinamidase, and nicotinate can then be converted to 6-hydroxynicotinic acid via nicotinate dehydrogenase (Fig. 5C) (Huang et al., 2022). Untargeted metabolomics analysis showed that the relative abundance of nicotinamide gradually decreased with storage time. In contrast, nicotinate and 6-hydroxynicotinic acid continuously accumulated. The changes in these metabolites were consistent with the proposed pathway of nicotinamide conversion to 6-hydroxynicotinic acid via nicotinate.

### 3.5 Targeted quantification of 4-hydroxyphenethyl alcohol and 6-hydroxynicotinic acid

Based on the untargeted metabolomics analysis, two candidate biomarkers, 4-hydroxyphenethyl alcohol and 6-hydroxynicotinic acid, were identified, and their MS/MS mirror spectra are provided in Supplementary Fig. S3. To verify the absolute concentration changes of the two metabolites, a targeted quantitative method based on UPLC–QQQ–MS/MS was established in this study (Fig. S4A–D). Both 4-hydroxyphenethyl alcohol and 6-hydroxynicotinic acid exhibited good linearity, with coefficients of determination (R^2^) greater than 0.999. The LOD and LOQ for 4-hydroxyphenethyl alcohol were 66.67 and 133.33 μg/kg, respectively, while those for 6-hydroxynicotinic acid were 0.67 and 1.67 μg/kg, respectively (Fig. 6A–B and Table S2). The targeted quantification results showed that the contents of 4-hydroxyphenethyl alcohol at D7 and D11 reached 1025.69 and 5081.85 μg/kg, respectively, while those of 6-hydroxynicotinic acid were 685.18 and 1368.12 μg/kg, respectively. These results were highly consistent with the increasing trends observed in the untargeted metabolomics data (Fig. 6C–D). At the critical spoilage point (D7), the concentrations of both metabolites far exceeded their respective LOQ values. At the same time point, TVC exceeded 6 log CFU/g and TVB-N exceeded the national standard limit of 15 mg/100 g, indicating that the sharp accumulation of these two metabolites coincided with the key turning point of beef quality deterioration.

**Fig. 6:**
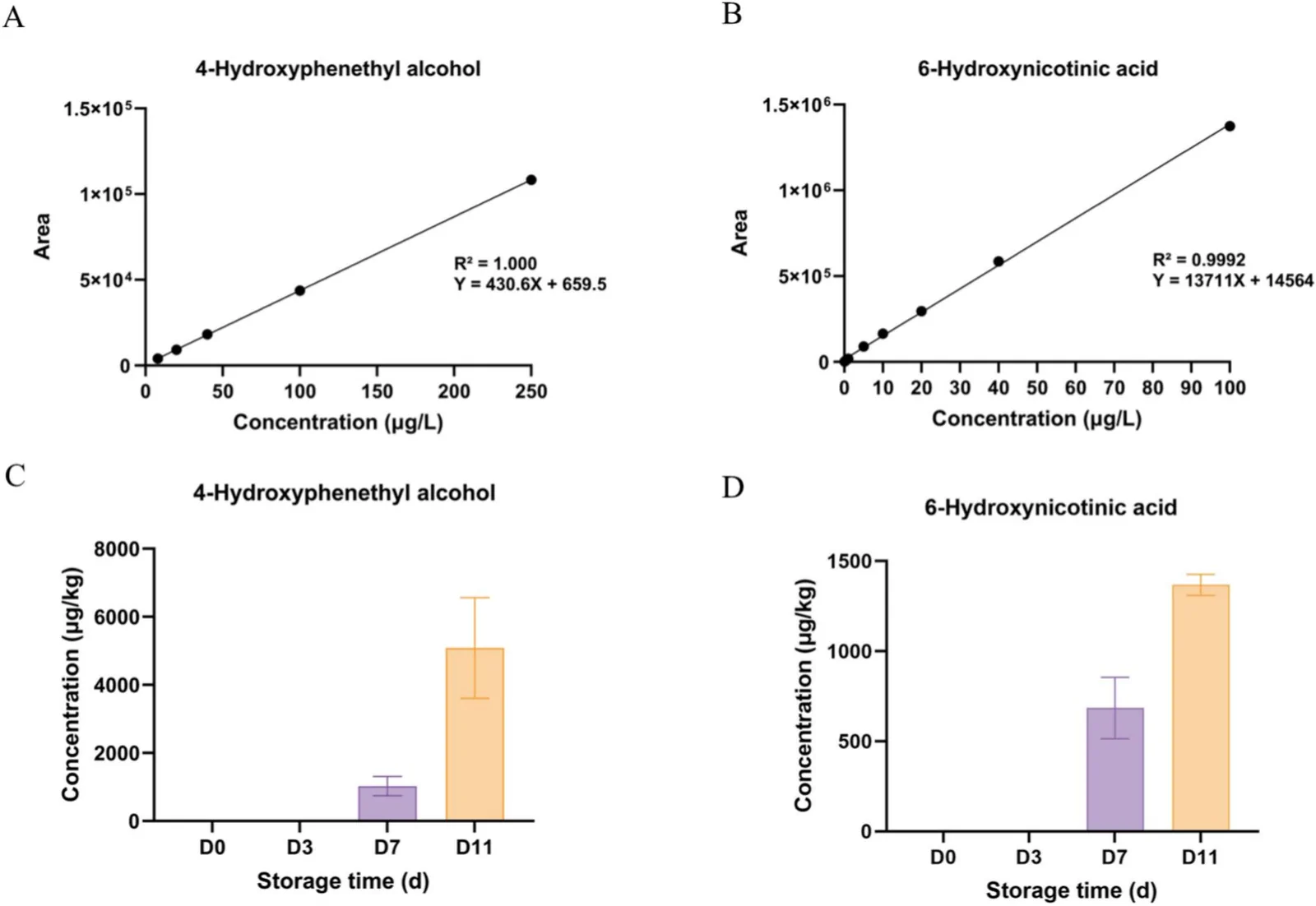
Calibration curves and quantitative analysis of selected metabolites in beef during refrigerated storage. (A) Calibration curve of 4-hydroxyphenethyl alcohol. (B) Calibration curve of 6-hydroxynicotinic acid. (C) Quantitative results of 4-hydroxyphenethyl alcohol in beef during refrigerated storage. (D) Quantitative results of 6-hydroxynicotinic acid in beef during refrigerated storage.

Traditional freshness evaluation indicators have certain limitations in practical applications. The reliability of TVB-N is greatly influenced by the composition of the dominant spoilage microbiota. For instance, when *Shewanella* predominates, volatile basic nitrogenous compounds are more readily formed during spoilage, and TVB-N corresponds well with the degree of spoilage. However, when *Pseudomonas* spp. are the dominant spoilage organisms, the evaluative efficacy of TVB-N is relatively limited (Hu et al., 2025). In addition, conventional TVC determination is time-consuming and labor-intensive, typically requiring 48–72 h to obtain results (Santovito et al., 2022). Traditional physicochemical analyses are limited in elucidating the underlying molecular mechanisms of meat spoilage. In the present study, 4-hydroxyphenethyl alcohol and 6-hydroxynicotinic acid exhibited more pronounced stage-specific accumulation characteristics during the transition from “fresh” to “spoiled”. Therefore, these two metabolites show promise as potential characteristic biomarkers for monitoring beef spoilage during refrigerated storage at 4 °C.

## 4 Conclusions

In this study, a strategy combining untargeted metabolomics screening with targeted quantitative validation was employed to screen and validate 4-hydroxyphenethyl alcohol and 6-hydroxynicotinic acid as two novel spoilage biomarkers during the refrigerated storage of beef at 4 °C. The untargeted metabolomics results showed that the relative abundances of 4-hydroxyphenethyl alcohol on days 7 and 11 reached 28.88 and 115.20 times that on day 0, respectively, whereas those of 6-hydroxynicotinic acid reached 33.47 and 66.66 times that on day 0, respectively. Targeted quantification showed that the contents of 4-hydroxyphenethyl alcohol on days 7 and 11 were 1025.69 and 5081.85 μg/kg, respectively, while those of 6-hydroxynicotinic acid were 685.18 and 1368.12 μg/kg, respectively. Overall, the sharp accumulation of 4-hydroxyphenethyl alcohol and 6-hydroxynicotinic acid coincided with the spoilage point at which TVC and TVB-N exceeded their threshold values, indicating their promising potential as spoilage indicators.

Based on the sharp increase in the concentrations of the two metabolites at the critical spoilage point, targeted rapid detection techniques, such as immunochromatographic test strips, electrochemical biosensors, or colorimetric kits, could be developed in the future for on-site real-time evaluation of beef freshness, thereby providing convenient and efficient new tools for quality monitoring in the meat supply chain. It should be noted that this study was conducted using beef from a single breed, and further research is needed to verify the representativeness of these biomarkers across a broader range of samples and to precisely trace the spoilage-related microbiota. Future studies should expand the sample size to include multiple breeds for targeted absolute quantification validation, and integrate microbiomics and functional gene analysis to refine the metabolic pathways and establish a multidimensional early-warning model for spoilage.

## CRediT authorship contribution statement

**Chuan Liu:** Writing – review & editing, Writing – original draft, Software, Methodology, Conceptualization. **Nan Liu:** Writing – original draft, Software, Methodology, Conceptualization. **Juan Zhang:** Formal analysis, Data curation. **Linlin Wang:** Investigation, Data curation. **Chunjiao Bao:** Investigation, Data curation. **Hao Liu:** Investigation, Formal analysis. **Wei Zhang:** Writing – review & editing, Methodology. **Lirong Sun:** Writing – review & editing, Resources, Methodology. **Ailiang Chen:** Writing – review & editing, Resources, Methodology.

## Declaration of competing interest

The authors declare that they have no known competing financial interests or personal relationships that could have appeared to influence the work reported in this paper.

## Data Availability

Data will be made available on request.

## Appendix A Supplementary data

Supplementary material: Figures S1–S4 and Tables S1–S2 supporting the metabolomics analysis and targeted quantification during refrigerated storage of beef.
