# Supplementary material for: Identification of 4-hydroxyphenethyl alcohol and 6-hydroxynicotinic acid as novel spoilage biomarkers in refrigerated beef
Source: Food Chem X. 2026 Sep 30;39:104549. doi: 10.1016/j.fochx.2026.104549 (PMC13660530; doi:10.1016/j.fochx.2026.104549)
Supplement: Supplementary file 1 [file mmc1.docx]

**Supplementary materials**

**Figure S1.** Permutation tests of the OPLS-DA models for (A) D3 vs. D0, (B) D7 vs. D0, and (C) D11 vs. D0 based on 1,000 permutations.

**Figure S2.** ROC curves of the 12 additional candidate metabolites evaluated in this study, including tyramine, Leu-Pro, Pro-Ile-Lys, p-cresol, nicotinate, xanthine, isonicotinic acid, Ile-Lys, Pro-Val, hypoxanthine, Asp-Trp, and Pro-Trp.

**Figure S3.** MS/MS mirror spectra of the two candidate biomarkers. (A) 4-Hydroxyphenethyl alcohol. (B) 6-Hydroxynicotinic acid.

 **Figure S4.** Representative ion chromatograms of 4-hydroxyphenethyl alcohol and 6-hydroxynicotinic acid obtained by UPLC–QQQ–MS/MS. (A) Chromatogram of the qualifier ion of 4-hydroxyphenethyl alcohol at m/z 106.200. (B) Chromatogram of the quantifier ion of 4-hydroxyphenethyl alcohol at m/z 119.000. (C) Chromatogram of the qualifier ion of 6-hydroxynicotinic acid at m/z 94.100. (D) Chromatogram of the quantifier ion of 6-hydroxynicotinic acid at m/z 122.100.

**Table S1.** Fold changes in differential metabolites during refrigerated storage of beef.

| Metabolite name | Fold Change (D3/D0) | Fold Change (D7/D0) | Fold Change (D11/D0) |
| --- | --- | --- | --- |
| 4-Hydroxyphenethyl alcohol | 1.883 | 28.884 | 115.200 |
| Tyramine | 2.291 | 57.060 | 90.865 |
| 6-Hydroxynicotinic acid | 3.359 | 33.471 | 66.657 |
| Malonic acid | 3.753 | 187.660 | 54.029 |
| 12-Hydroxyoctadecanoic acid | 0.427 | 33.031 | 48.812 |
| Nicotinate | 2.096 | 15.360 | 35.216 |
| 3-Oxostearic acid | 0.688 | 23.418 | 33.852 |
| trans-Vaccenic acid | 0.792 | 17.135 | 27.604 |
| 4-Hydroxydodecanoic acid | 0.763 | 5.418 | 27.589 |
| Cinnamic acid | 1.417 | 7.020 | 23.228 |
| Isonicotinic acid | 1.966 | 10.184 | 22.281 |
| Isopentenyladenine | 0.876 | 7.809 | 21.009 |
| DL-Leu-DL-Val | 0.770 | 5.846 | 19.100 |
| N2,N2-Dimethylguanosine | 1.447 | 8.982 | 17.233 |
| p-Cresol | 1.898 | 15.199 | 16.552 |
| D-(-)-Glutamic acid | 1.272 | 3.741 | 15.769 |
| Pro-Ile-Lys | 2.314 | 7.082 | 15.606 |
| 2-Keto-L-gulonic acid | 1.324 | 54.823 | 14.745 |
| 3-Phenyllactic acid | 1.166 | 4.470 | 14.466 |
| L-3-Aminobutyric acid | 1.437 | 4.142 | 13.721 |
| L-Glutamic acid | 1.239 | 3.539 | 11.421 |
| PC(15:0/0:0) | 0.704 | 2.088 | 8.601 |
| Riboflavin | 1.539 | 2.575 | 8.517 |
| 8-(3-Octyl-2-oxiranyl)octanoic acid | 0.934 | 5.662 | 8.175 |
| PE(20:4/0:0) | 0.721 | 2.300 | 7.080 |
| Aspartic acid | 1.416 | 5.009 | 6.994 |
| Pro-Thr | 1.941 | 5.284 | 6.528 |
| Pro-Val | 1.776 | 3.800 | 6.496 |
| 2-Dimethylamino-6-hydroxypurine | 1.092 | 2.326 | 6.467 |
| 6-Oxooctadecanoic acid | 1.144 | 4.716 | 6.435 |
| Ile-Lys | 1.626 | 2.811 | 6.269 |
| Pro-Leu | 1.315 | 4.266 | 6.195 |
| Asp-Trp | 1.830 | 3.694 | 6.157 |
| PC(0:0/20:4) | 0.633 | 2.386 | 6.056 |
| L-2-Hydroxyglutaric acid | 2.662 | 7.250 | 5.961 |
| Pro-Trp | 1.609 | 3.227 | 5.786 |
| N6-Threonylcarbamoyladenosine | 1.254 | 3.610 | 5.561 |
| 6-Hydroxyoctadec-4-enoic acid | 0.666 | 3.179 | 5.411 |
| Ile-Pro | 1.430 | 2.619 | 5.342 |
| Val-Pro | 1.697 | 2.703 | 5.273 |
| 7-Methylguanine | 1.206 | 3.923 | 5.244 |
| Methyl hexadec-9-enoate | 0.966 | 3.533 | 5.104 |
| 9,10-Epoxystearic acid | 0.518 | 2.995 | 5.011 |
| Linoleic acid | 0.808 | 3.074 | 4.975 |
| 5'-Methylthioinosine | 2.152 | 7.317 | 4.875 |
| N-Acetyl-L-carnosine | 1.388 | 2.199 | 4.867 |
| Leu-Pro | 1.651 | 2.953 | 4.793 |
| His-Arg-Lys | 0.857 | 4.969 | 4.784 |
| Arachidonic acid | 1.455 | 3.064 | 4.705 |
| Pro-Phe | 1.478 | 3.679 | 4.638 |
| Val-Glu | 1.784 | 3.131 | 4.624 |
| Hypoxanthine | 1.847 | 2.919 | 4.542 |
| Ile-Thr | 1.466 | 2.726 | 4.483 |
| Trp-Pro | 1.355 | 2.057 | 4.451 |
| Trp-Glu | 1.481 | 2.738 | 4.176 |
| Linoelaidic acid | 0.782 | 2.499 | 4.168 |
| His-Phe | 1.417 | 3.150 | 4.062 |
| PC(18:2/0:0) | 0.639 | 1.831 | 4.012 |
| Val-Val | 1.396 | 2.932 | 3.961 |
| D-Sedoheptulose-7-phosphate | 1.153 | 2.072 | 3.587 |
| Benzaldehyde | 1.150 | 3.282 | 3.520 |
| Asp-Leu | 1.609 | 3.851 | 3.482 |
| 6-Phosphogluconic acid | 1.138 | 1.333 | 3.479 |
| Xanthine | 1.632 | 2.545 | 3.357 |
| Citrulline | 0.932 | 3.920 | 3.305 |
| Ala-Trp | 0.631 | 1.448 | 3.237 |
| Glu-Val | 1.291 | 2.037 | 3.186 |
| Glu-Trp | 0.713 | 1.699 | 3.154 |
| Indole-3-acetate | 1.066 | 1.810 | 3.136 |
| Trp-Gln | 0.857 | 1.666 | 3.026 |
| D-Glucosamine 6-phosphate | 1.420 | 1.653 | 2.959 |
| Phe-Thr | 0.953 | 1.395 | 2.790 |
| Indole-3-carboxyaldehyde | 1.037 | 1.832 | 2.755 |
| His-Leu | 1.069 | 1.814 | 2.647 |
| L-Norvaline | 1.093 | 1.735 | 2.647 |
| Pro-Glu | 0.973 | 1.289 | 2.617 |
| Gly-Phe | 0.986 | 1.673 | 2.576 |
| Phe-Gly | 0.949 | 1.882 | 2.520 |
| Phe-Asn | 1.112 | 1.593 | 2.488 |
| Val-Tyr | 1.063 | 1.692 | 2.453 |
| Tryptophan | 1.074 | 1.896 | 2.446 |
| Proline | 1.374 | 1.655 | 2.437 |
| Glycerol 1-myristate | 0.591 | 1.323 | 2.419 |
| Val-Asp | 1.357 | 2.091 | 2.396 |
| PyroGlu-Trp | 0.769 | 1.429 | 2.392 |
| L-Isoleucine | 1.212 | 1.756 | 2.372 |
| L-Leucine | 1.082 | 1.715 | 2.369 |
| Phenylalanine | 1.051 | 1.732 | 2.344 |
| Glycyl-L-norleucine | 1.038 | 1.418 | 2.334 |
| LPC 18:2 | 0.505 | 0.941 | 2.326 |
| Palmitelaidic acid | 0.698 | 0.992 | 2.284 |
| Tyr-Gly | 1.142 | 1.604 | 2.279 |
| 14-Hydroxymyristic acid | 0.831 | 1.655 | 2.260 |
| Ile-Phe | 0.840 | 1.251 | 2.218 |
| Tyr-Glu | 1.545 | 2.087 | 2.208 |
| Xanthosine | 1.732 | 1.471 | 2.195 |
| His-Ile | 1.037 | 1.655 | 2.171 |
| Trp-Lys | 0.969 | 1.455 | 2.169 |
| 3-OH-isovaleric acid | 1.015 | 1.212 | 2.139 |
| Leu-His | 1.080 | 1.683 | 2.096 |
| Guanosine | 1.318 | 1.597 | 2.057 |
| Phe-Gln | 0.954 | 1.349 | 2.027 |
| Gly-Leu | 1.164 | 1.728 | 2.010 |
| Indole | 0.958 | 1.522 | 2.008 |
| L-Methionine | 1.055 | 1.793 | 2.006 |
| DL-Indole-3-lactic acid | 0.937 | 1.123 | 1.981 |
| Glu-Tyr | 1.242 | 2.104 | 1.972 |
| Ile-Met | 1.114 | 1.304 | 1.905 |
| DL-Phenylalanine | 1.159 | 1.559 | 1.793 |
| Creatinine | 1.148 | 1.436 | 1.754 |
| AICA-riboside | 1.288 | 1.605 | 1.726 |
| Hemin cation | 2.156 | 1.190 | 1.722 |
| 4-Pyridoxic acid | 1.913 | 2.319 | 1.715 |
| 2-Amino-1-phenylethanol | 1.151 | 1.491 | 1.689 |
| D-Tryptophan | 1.175 | 1.543 | 1.686 |
| trans-Cinnamic acid | 1.137 | 1.455 | 1.642 |
| 3-Phenoxypropionic acid | 1.129 | 1.428 | 1.630 |
| Asp-Ile | 1.699 | 1.715 | 1.616 |
| Maleic acid | 0.926 | 0.591 | 1.549 |
| Uric acid | 1.140 | 1.620 | 1.453 |
| N-Acetyl-L-methionine | 0.992 | 0.610 | 1.209 |
| Hexanoyl-L-carnitine | 0.664 | 0.518 | 0.836 |
| Phe-Arg | 0.700 | 0.428 | 0.726 |
| Cys-Phe | 0.807 | 0.644 | 0.663 |
| n-Glycerol 3-phosphate | 0.350 | 0.087 | 0.638 |
| Ac-Asp-Glu | 1.065 | 0.631 | 0.593 |
| Hippurate | 1.034 | 0.621 | 0.576 |
| D-Ribulose 5-phosphate | 0.841 | 0.733 | 0.539 |
| 1-Pentadecanoyl-sn-glycero-3-phosphocholine | 0.668 | 0.632 | 0.484 |
| Lauroylcarnitine | 0.529 | 0.394 | 0.379 |
| Uridine | 1.424 | 1.099 | 0.356 |
| Adenosine 2'-monophosphate | 0.661 | 0.667 | 0.353 |
| Lauryldiethanolamine | 0.609 | 0.715 | 0.352 |
| Adenosine | 0.694 | 0.487 | 0.351 |
| L-Tyrosine | 1.218 | 0.716 | 0.343 |
| p-Hydroxyphenyllactic acid | 1.192 | 0.685 | 0.340 |
| Octanoylcarnitine | 0.273 | 0.365 | 0.296 |
| Ribulose 5-phosphate | 0.773 | 0.791 | 0.294 |
| Nicotinamide | 0.964 | 0.621 | 0.249 |
| Myo-inositol | 0.887 | 0.408 | 0.208 |
| L-Sorbose | 0.907 | 0.420 | 0.199 |
| Decanoylcarnitine | 0.406 | 0.357 | 0.198 |
| L-Arachidonoylcarnitine | 0.586 | 0.397 | 0.184 |
| Linoleoylcarnitine | 0.490 | 0.380 | 0.179 |
| Stearoyl-L-carnitine | 0.300 | 0.213 | 0.178 |
| Adenylosuccinic acid | 0.980 | 0.770 | 0.172 |
| Arginine | 1.185 | 0.554 | 0.151 |
| D-(-)-Isoascorbic acid | 0.093 | 0.181 | 0.147 |
| 3'-Dephosphocoenzyme A | 0.602 | 0.370 | 0.144 |
| N-Acetyl-D-glucosamine 6-phosphate | 0.915 | 0.318 | 0.114 |
| Palmitoylcarnitine | 0.150 | 0.112 | 0.111 |
| D-Desthiobiotin | 0.105 | 0.090 | 0.093 |
| Oleoyl-carnitine | 0.165 | 0.108 | 0.088 |
| Succinoadenosine | 0.867 | 0.314 | 0.061 |
| S-(5'-Adenosyl)-L-homocysteine | 1.058 | 0.275 | 0.055 |
| 3-Hydroxyoleylcarnitine | 0.187 | 0.094 | 0.051 |
| Uridine-5-monophosphate | 0.637 | 0.159 | 0.027 |
| L-Glutamine | 1.002 | 0.354 | 0.014 |
| Inosine 5'-monophosphate | 0.702 | 0.264 | 0.004 |

**Table S2.** Standard curves, Linear ranges, LODs, and LOQs of the target compounds.

| Metabolite name | Standard curves | R^2^ | Linear ranges (µg/L) | LOD (μg/kg) | LOQ (μg/kg) |
| --- | --- | --- | --- | --- | --- |
| 6-Hydroxynicotinic acid | Y = 13711X + 14564 | R² = 0.9992 | 0.1-100.0 | 0.67 | 1.67 |
| 4-Hydroxyphenethyl alcohol | Y = 430.6X + 659.5 | R² = 1.000 | 8.0-250.0 | 66.67 | 133.33 |
